# All around the Year 1893

**Published:** 1892-12

**Authors:** 


ALL AROUND THE YEAR 1893. Lee & Shepard: 10 Milk Street, Boston, Mass. Entirely new design in colors, by J. Pauline Sunter. Printed on heavy card-board, gilt edges, with chain, tassels, and ring. Size, $4\frac{1}{4}$x$5\frac{1}{2}$ inches. Boxed. Price, 50 cents.

The "All Around the Year" calendar which Mrs. Sunter sends out this year is as charming a piece of work as anything she has done. Like its predecessors, it is printed on heavy card-board, gilt-edged, with chain, tassels, and ring, and is of convenient size. The designs are fresh and delightful, quaint and picturesque little lads and lasses issuing in each month with just the right words, and in the most charming attitudes, while the lines on the cards combine to form a very pleasing love story. Done in several colors, one can scarcely imagine anything more graceful than the twelve cards, each bearing the dainty design which includes the month's calendar as a part of the picture. The cover shows a pretty little Miss watching a Cupid "warming his pretty little toes" at an open fireplace, while on the last page this same Cupid (or his fellow) is playing sweetly, "Good-bye, My Lover, Good-bye."

